# RNA-Based Therapeutic Technology

**DOI:** 10.3390/ijms242015230

**Published:** 2023-10-16

**Authors:** Ryuichi Mashima, Shuji Takada, Yoshitaka Miyamoto

**Affiliations:** 1 Department of Clinical Laboratory Medicine, National Center for Child Health and Development, 2-10-1 Okura, Setagaya-ku, Tokyo 157-8535, Japan; 2 Department of Systems BioMedicine, National Research Institute for Child Health and Development, 2-10-1 Okura, Setagaya-ku, Tokyo 157-8535, Japan; 3 Department of Maternal-Fetal Biology, National Research Institute for Child Health and Development, 2-10-1 Okura, Setagaya-ku, Tokyo 157-8535, Japan

**Keywords:** LNP, immunotherapy, U7 snRNA, ADAR

## Abstract

RNA-based therapy has been an expanding area of clinical research since the COVID-19 outbreak. Often, its comparison has been made to DNA-based gene therapy, such as adeno-associated virus- and lentivirus-mediated therapy. These DNA-based therapies show persistent expression, with maximized therapeutic efficacy. However, accumulating data indicate that proper control of gene expression is occasionally required. For example, in cancer immunotherapy, cytokine response syndrome is detrimental for host animals, while excess activation of the immune system induces supraphysiological cytokines. RNA-based therapy seems to be a rather mild therapy, and it has room to fit unmet medical needs, whereas current DNA-based therapy has unclear issues. This review focused on RNA-based therapy for cancer immunotherapy, hematopoietic disorders, and inherited disorders, which have received attention for possible clinical applications.

## 1. Introduction

Gene therapy has now been accepted as a novel therapeutic technique [1,2]. Such applications include cancer immunotherapy [3] and the treatment of hematopoietic disorders [4] and inherited diseases [5]. First, it was more than 10 years ago that CD19-targeting chimeric antigen receptor (CAR) T cell therapy was originally reported to be effective for B cell lymphoma [3]. This therapy expresses therapeutic cDNA harboring a single chain variable fragment that specifically recognizes CD19, a costimulatory domain of 4-1BB, and a cytoplasmic domain of CD3ζ that contains immunoreceptor tyrosine-based activation motif (ITAM). Based on the initial success of CD19 as a target for gene therapy, the potential for NKG2D, a receptor for natural killer (NK) cells, was also examined. Second, β-thalassemia is caused by a deficiency or impaired expression of the β-hemoglobin gene, causing severe anemia. Gene supplementation of its functional form is effective for transfusion-free life in patients [4,6]. Third, spinal muscular atrophy (SMA) is a muscle disorder in neonates and children that loses the functional SMN1 gene at the telomeric chromosome 5. In humans, there is a centromeric SMN2 gene harboring C>T mutation at exon 7, making SMN2 mRNA inactive due to out-of-frame mutation. Based on this mechanism, an approved therapy produces an active SMN2 transcript through exon inclusion with adeno-associated virus (AAV) or a small molecule-mediated strategy [5].

There are two best-characterized gene delivery techniques. First, the lentiviral vector is used for gene delivery to hematopoietic stem cells. For administration, hematopoietic cells are removed from the body by apheresis and then infected with lentivirus ex vivo. There is no limitation to the size of the cDNA. After infection, these cells are infused back into the patient’s body. Due to such clinical procedures, there are a limited number of medical facilities that only perform this procedure. Lentivirus is capable of infecting non-growing cells. Second, the adeno-associated virus is another widely used vector. This particle has an icosahedral shape made of several structural proteins involving VP1, VP2, and VP3 [7]. The vector has a unique sequence called inverted terminal repeats (ITR) at both the 5′ and 3′ ends. The size of the payload is limited to 4.0–4.8 kb in AAV. There are nine serotypes known for humans. In addition, some serotypes for monkeys can infect humans, leading to a wider choice of clinical applications. In addition, there are some engineered serotypes that are not present in nature. One advantage of AAV over lentiviral vectors may be linked to direct administration through infusion. Furthermore, AAV can be infected with nondividing cells. The benefit of AAV-based gene therapy is that it only requires one-time treatment in life. Recently, therapeutic AAV for SMN has been approved [8,9,10]. A current concern for these vectors relates to a chance of DNA insertion that might cause genotoxicity for lentiviral vector and hepatotoxicity for AAV, respectively.

An increasing number of studies have suggested that viral-free gene therapy has been appreciated [11,12,13] (Table 1). For RNA-based technology, nanoparticle-based technology, electroporation, and vector-based technology under RNA polymerase specific promoter are widely used. A previously developed technique often used vector-based technology, partly because therapeutic RNA was unmodified and unstable in vivo. However, current technology uses a modified nucleoside, such as N1-methypseudouridine, for a better therapeutic outcome with lesser interferon production. Lipid nanoparticle (LNP) is a vector-free gene delivery system. It mainly targets the liver. Due to several limitations for clinical application, LNP still has room for innovation as a gene delivery tool. However, there are some interesting clinical applications, as described in this review. LNP has been used as an encapsulating agent for Onpattro, an siRNA drug for amyloidosis [14]. The COVID-19 vaccine was similarly formulated by LNP based on this experience [15]. A transposase-mediated production of CAR-T cells by electroporation is another active research area for a viral-free DNA delivery technique [16]. In this narrative review, gene delivery techniques and payloads for mRNA are reviewed.

## 2. Gene Delivery Techniques for mRNA

### 2.1. LNP

LNP is a nanoparticle made of four species of lipids [44]. These include ionizable cationic lipids, cholesterol, helper phospholipids, and PEG lipids. Cholesterol determines the physical properties of the LNP. The content of ionizable cationic lipids determines the efficiency of payload release. PEG lipids are linked to the hydrophilicity of LNP, which is associated with the stability of LNP in water. LNP is an emerging technique for RNA therapy with a gradual increase in applications.

Several studies have reported the efficacy of antibody-mediated tissue-specific targeting of LNP. One successful example is N-succinimidyl S-acetylthioacetate (SATA)-maleimide chemistry [45]. In this reaction, micelles containing a PEG-lipid derivative functionalized with maleimide (DSPE-PEG-mal) are mixed with already formed mRNA-LNPs to insert DSPE-PEG-mal into the LNPs. The antibodies are then functionalized through the introduction of protected sulfhydryl groups on primary amines using SATA. After deprotection, antibodies are conjugated to LNPs through a thioether bond between the sulfydryl and maleimide groups. With this approach, mRNA was delivered specifically to PECAM-1 [46], CD4 [47], and CD5-positive cells [11]. The best-described example involves anti-CD5 antibody-mediated cardiomyocyte-specific targeting [11]. More recently, anti-CD117 antibody-mediated hematopoietic stem cell–specific targeting was reported [12,48].

Selective organ targeting (SORT) is a recently proposed hypothesis that is achieved by optimizing the lipid species and composition of LNP [49,50]. It is known that prototypical LNP targets the liver [44]. This is based, at least in part, on the apoE-dependent mechanism. This liver-specific targeting could be modulated to either the spleen or lung by adding a fifth lipid component, such as 18PA for the spleen and DOTAP for the lung [50]. Alternatively, targeting the spleen involves endothelial-specific receptors, such as PECAM-1 [46]. Whether both play a key role in LNP incorporation might be affected by its expression level.

Specific tissue targeting the brain and bone is still a challenging area. Penetration of the blood–brain barrier (BBB) has been considered an issue to be resolved. This is due to pericytes, a subtype of endothelial cells, surrounding brain vessels. These cells line very tightly along blood vessels, preventing material transportation from the blood to the brain. A recent study by Saha et al. reported an amphetamine-decorated LNP that enhanced BBB penetration [51]. In this case, the penetration of the BBB would be expected by the simultaneous endocytosis of a complex with the adrenaline receptor. Similarly, the delivery of drugs to the bone microenvironment is another area of investigation. Xue et al. reported the possibility of bisphosphonate-modified LNP [52]. The administration of BMP2 mRNA in vivo stimulated its expression in the tibia in a mouse model.

### 2.2. Electroporation

Electroporation is another widely used technique for gene delivery to immune cells, such as T cells [53] (Table 1). Its idea of utilizing in vitro transcribed (IVT) mRNA as a therapeutic agent goes back to the 1980s; progress was hindered by challenges, such as low stability and immunogenicity in vivo. However, the advent of nucleoside-modified mRNA technology has notably diminished mRNA’s immunogenicity while enhancing its translation efficiency, accelerating the advancement of mRNA therapeutics. The disadvantage of electroporation is its deteriorating effect on cells; however, improved electroporation conditions have been developed. In fact, a recent study reported that the efficiency of gene deletion to T cells by LNP, electroporation, and lentiviral transduction becomes comparable in vitro [54].

## 3. Payloads

### 3.1. Therapeutic cDNA

Expression of therapeutic cDNA is the first choice for gene therapy [55,56]. Based on accumulating data, codon optimization, capping, or synthetic modification of several residues of ribonucleosides at the 5′ and 3′ terminal, and replacement of some ribonucleosides, such as uridine to N1-methylpseudouridine, are known to increase the stability of RNA in vivo. To minimize interferon production, the synthesized mRNA might be treated with cellulose to eliminate trace amounts of dsRNA [57].

#### 3.1.1. CAR

##### CAR-T

CARs have shown exceptional promise in clinical applications [58]. The first-generation CAR has a single chain variable fragment for CD19 and a single CD3ζ intracellular signaling domain with no costimulatory domain. Second-generation CAR has a costimulatory domain of either CD28 or 4-1BB. Third-generation CAR has two or more costimulatory domains with enhanced cytokine production and tumor-killing activity. Fourth-generation CAR is designed to secrete cytokines in response to antigen stimulation.

For functional improvement of CAR-T cells, a comparison of the costimulatory domain from CD28 and 4-1BB is under active discussion [59,60]. The activation of the T cell receptor induces tyrosine phosphorylation of ITAM. Under this stimulation condition, CD28-, but not 4-1BB-, based CAR only recruits Lck to its ITAM [58]. Interestingly, CD28-based CAR can be activated rapidly, but it also exhausts rapidly and no longer persistently transduces intracellular signaling. In contrast, upon ITAM activation in 4-1BB-based CAR, a unique FOXO3-mediated dysfunction profile was observed [61]. Currently approved agents, such as Yescarta and Tecartus, use the CD28-based costimulatory domain, while Kymriah and Breyanzi use the 4-1BB-based costimulatory domain. The difference between these CARs further depends on their hinge and transmembrane domain. Their influence ultimately affects their therapeutic efficacy with many variables.

##### CAR-NK

CAR-NK has attracted attention for anticancer therapy, particularly for solid tumors [62]. CAR-NK cells may serve as alternative candidates for retargeting cancer because of their unique recognition mechanisms, powerful cytotoxic effects, especially on cancer cells in both CAR-dependent and CAR-independent manners, and clinical safety. Essentially, the target cell recognition method for NK cells is MHC class I independent. Rather, killer Ig-like receptors (KIR) in NK cells can inhibit the activation of NK cells through MHC-class-I-mediated interaction. Furthermore, cytokine profiles released from NK cells are unique to IFN-γ and GM-CSF, rather than that of the cytokine release syndrome of CAR-T therapy, where IL-1, IL-6, IL-10, and TNF-α are predominant. Thus, NK cells can serve as an “off-the-shelf product” because NK cells from allogeneic sources can also be used in immunotherapies owing to their reduced risk of alloreactivity.

An earlier study reported that the expression of the extracellular domain of NK cell receptor NKG2D to an intracellular adaptor DAP-12 increased NK-cell-mediated tumor-killing activity [63]. Separately, Wilk et al. demonstrated the possibility of CAR-NK cells by mRNA using charge-altering releasable transporters [32].

##### CAR-M

Macrophages have recently emerged as prominent candidates for the treatment of solid tumors [64]. Macrophages are innate immune cells that are intrinsically equipped with broad therapeutic effector functions, including active migration to tumor sites, tumor phagocytosis, activation of the tumor microenvironment, and professional antigen presentation. Thus, the generation of CAR macrophages (CAR-M) is an expected area of research. Ye et al. reported the generation of CD19-CAR-expressing macrophages using LNP and further demonstrated tumor-killing activity in vitro [65]. These data demonstrate that optimized LNP composition allows gene delivery to macrophages.

#### 3.1.2. Cancer Immunotherapy

Cancer immunotherapy is another promising area for this application. Encapsulated LNPs that target the lymph node activate dendritic cells through T cells. mRNA that encode DC-stimulating peptides presented by T cells can be synthesized by mRNA. Thus, this system efficiently activates peripheral immunity. Compared to typical CAR-mediated hematologic therapy, LNP-based therapy does not require ex vivo gene delivery. In fact, an idea for DC activation has been examined using peptides rather than mRNA [66]. However, such a previous method was not established because of a higher immune response. Current mRNA-based technology has produced smaller concentrations of interferons and cytokines so that the interaction between T cells and DCs could be maximized. Ongoing clinical trials seem to be largely involved in malignant tumors.

The UK–BioNTech partnership for mRNA cancer vaccines is a recently launched project that plans to produce 10,000 mRNA vaccines for personalized immunotherapy by 2030 [67]. BioNTech, a German company initially founded in 2008, aims to develop personalized mRNA-based cancer therapy. The efficacy of this therapy has been partly proved by the results of a Phase I study of a combination study of atezolizumab + chemotherapy and a cancer mRNA vaccine for patients with pancreatic ductal adenocarcinoma. This new project also includes the identification of tumor neoantigens that have individually different private mutations. According to the UK government, the first trial is scheduled for September 2023.

### 3.2. Cas9, Base Editors, and Prime Editors

Cas9 nuclease and its derivatives, such as base editors and prime editors, are also widely chosen for the payload of LNP [68]. Cas9-mediated genome editing undergoes two distinct mechanisms: nonhomologous end-joining (NHEJ) and homology-directed repair (HDR). The adenine base editor produces inosine from adenine, leading to A-to-G conversion. Similarly, the cytidine base editor produces cytidine to uracil, leading to C-to-T conversion. Apart from these deaminase-mediated base editings, the prime editor uses reverse transcriptase. In this case, upon binding of modified Cas9 to the PAM-directed genome sequence, a reverse transcriptase fused to this modified Cas9 synthesizes an edited genome based on template RNA. At this stage, the efficiency of gene correction of the former is known to be better than that of the latter. An advantage of the latter involves the replacement of genomes without genome excision [68].

Breda et al. recently reported that anti-CD117 antibody-conjugated LNPs efficiently penetrate hematopoietic stem cells [12] (Table 2). In this study, the authors focused on correcting pathogenic sickle cell disease using an adenine base editor. Using their optimized gRNA sequence, the number of sickle cells was significantly reduced in the mouse model. Furthermore, the delivery of p53 up-regulated modulator of apoptosis (PUMA) mRNA with CD117/LNP affected the function of hematopoietic stem cells (HSCs) and permitted a nongenotoxic conditioning regimen for hematopoietic stem cell transplant. Altogether, the ability to target HSCs in vivo offers a nongenotoxic conditioning regimen for hematopoietic stem cell transplants, and this platform could be the basis for in vivo genome editing to cure genetic disorders, which would abrogate the need for it.

### 3.3. Short Length RNA (siRNA and Antisense Oligonucleotide)

The success of Onpattro has been extensively reviewed [73]. This is an LNP-encapsulated siRNA drug that is effective for transthyretin amyloidosis [14]. For a shorter payload, the composition of LNP may be optimized with a reduced percentage of cationic ionizable lipids. For longer payloads, an increasing cationic lipid is required because the phosphate-derived negative charge becomes too high.

There have been some studies on siRNA expression toward inflammatory cytokines. This is the most effective method of immunotherapy. For example, Zhou et al. reported that the co-encapsulation of siRNA for IL-6 was effective for tumor eradication [18]. Cytokine-related syndrome (CRS) is a pathogenic condition in which the immune system responds to infection or immunotherapy drugs more aggressively than it should [45]. This is particularly obvious when viral-vector-mediated immunotherapy is performed. To overcome this clinical problem, a recent trend in the vector development of immunotherapy has shifted to electroporation or LNP. In some cases, an miR-122-based strategy has been examined for the reduction of hepatotoxicity [74].

In 2015, Nishina et al. reported that the DNA/RNA heteroduplex oligonucleotide effectively suppressed gene expression [75]. In that study, the authors first examined the suppression of ApoB mRNA through the administration of this DNA/RNA hetero duplex oligonucleotide in a murine LDL receptor-deficient mouse model. The study was then expanded to a cynomolgus monkey model. Interestingly, these authors mentioned that conjugation of α-tocopherol, a lipid-soluble antioxidant, efficiently enhances gene suppression. An improvement in stability by LNP encapsulation could be expected.

### 3.4. U7 snRNA

Uridine-rich small nuclear ribonucleoproteins (snRNPs) are complexes involved in the splicing of pre-mRNA [76,77,78,79]. U7 snRNA is a unique snRNA that is specifically involved in the formation of a complex with histone and pre-mRNA. The presence of U7 snRNA enhances the formation of the histone cleavage complex by recruiting a stem loop binding protein [80]. Essentially, some splicing events are regulated by cis-acting factors. In other words, splicing regulatory (SR) proteins bind to exonic and intronic splicing enhancers (ESE and ISE). In contrast, heteronuclear RNPs (hnRNPs) bind to the sequence of exonic and intronic splicing silencers (ESS and ISS) of the target genome. Therefore, U7 snRNA harboring antisense oligonucleotides to these sequences could manipulate the expression of pre-mRNA. Whether the sequence of interest may be enhanced or silenced may be predicted by in silico programs, such as the human splicing finder system [81].

Based on this mechanism, gene therapy methods for a variety of disorders, such as Duchenne muscular dystrophy (DMD) [82,83,84,85,86,87], β-thalassemia [88], SMA [89], Pompe disease [90], and others, have been examined (Table 3). In DMD, the loss of several exons becomes pathogenic due to out-of-frame splicing, so that in-frame exon inclusion can correct pathogenesis. In SMA, a loss of the telomeric SMN1 gene causes pathogenic conditions. SMN2, an SMN1-like gene with a C>T mutation in exon 7, is constitutively expressed while it is nonfunctional. For therapeutic purposes, exon inclusion of SMN2 is effective because this treatment makes the treated SMN2 protein functional.

Recently, Van der Wal et al. described a strategy for Pompe disease through gene therapy using splicing correction [90]. The authors focused on the correction of a common α-glucosidase (GAA) mutation: c.-32-13T>G. In this case, a mutant generates a normal transcript involving exons 1 through 3, as well as two distinct abnormal ones. One abnormal transcript involves exons 1 and 3, leading to a complete loss of exon 2. Another abnormal transcript involves partial exon 2, where its 3′ portion is linked to exons 1 and 3. These authors found many intron splicing silence sequences in this genome in silico, leading to the identification of key intron splicing enhancers in GAA intron 1. Using patient-derived fibroblasts, an increase in GAA enzyme activity was observed when these cells were treated with an exogenous antisense oligonucleotide.

### 3.5. Circular RNA

Circular RNA is an endogenously detectable minor fraction of RNA formally classified as noncoding RNA, but later it was found that this circular RNA was also detectable at a similar concentration to siRNA or miRNA [116]. Its formation has multiple mechanisms. Under normal splicing conditions, splicing of pre-mRNA occurs through the formation of a lariat structure, of which the 5′ end of the intron is circularized to its branching point as its intermediate structure, continuing sequentially from the last exon to exon 1 [116]. In contrast, a mechanism of circular RNA formation involves the back splicing of pre-mRNA, where the 5′ end of the exon links to the latter exon.

Adenosine deaminase acting on RNAs (ADAR) is an enzyme involved in the correction of adenine to inosine in mRNA [117]. In humans, there are several ADAR isozymes. ADAR1 p150 is a ubiquitous form of enzyme with full-length protein localizing in the cytoplasm, while ADAR1 p110 is a short form localizing in the nucleus. Functionally, ADAR p110 lacks a Zα. Therefore, the RNA-recognition domain does not have deaminase activity. Mutations in the ADAR1 gene cause Aicardi–Goutières syndrome (AGS), an infant encephalopathy with type I interferon (IFN) overproduction [118,119]. ADAR2 has been considered an inactive form. ADAR3 is found in the brain and is considered an important enzyme for mRNA maturation. A well-described example involves the serotonin 5HT2c receptor in the brain [120]. In this case, five adenosines of amino acid positions 156, 158, and 160 can be edited in mature mRNA. As a result, at most, three alterations of amino acids, such as isoleucine to valine at amino acid position 156, asparagine to glycine at amino acid position 158, and isoleucine to valine at amino acid position 160, occur, respectively. Interestingly, there is a family of 5HT2 receptors, such as 5HT2a and 5HT2b, with similar pre-mRNA sequences to 5HT2c, though these were not susceptible to RNA editing. Another involvement of ADAR in the pathogenesis has also been suggested in cancer [121]. Based on these examples, ADAR’s catalytic activity seems to be reasonably high, raising the possibility that its endogenous enzyme activity could be used for therapeutic applications [117,122].

Accumulating evidence has indicated that circularized gRNA for ADAR has extended stability in cells compared to linearized RNA [123,124]. The advantage of circular RNA involves lower immunogenicity and less susceptibility to exonucleases [117,125]. In a mouse model, one method uses ribozyme, a short RNA sequence with autocatalytic activity, to generate circularized RNA [126,127]. Another method uses a self-splicing intron with a rationally designed accessary sequence [128]. Its efficacy in vivo was demonstrated by a mucopolysaccharidosis type I mouse model [129]. In this experiment, circular RNA was expressed under the U6 promoter in AAV8 (Table 4). For the experiment in vitro, circular RNA needs HPLC purification for the best results at this stage. However, once prepared, LNP can encapsulate circular RNA with other payloads for gene delivery.

## 4. Conclusions and Future Perspectives

Gene therapy is a promising therapy with an increasing number of newly developed technologies. Currently, many clinical trials are ongoing. There are several concerns about gene therapy. One of the most important concerns involves medical economics. The average cost of AAV or lentiviral therapeutic agents exceeds more than USD 1 million. Therefore, in some cases, therapeutic expenses could be reimbursed depending on their efficacy (https://www.axios.com/2022/08/18/fda-gene-therapy-approval-zynteglo, accessed on 31 August 2023). There seems to be an opportunity to overcome this issue through the RNA-based vector free gene delivery method.

Newborn screening is a social platform that allows the detection of affected individuals at an early stage of life [130]. The number of applicable disorders may vary by country because the policies of authorized agencies and the frequency of affected individuals are not uniform. It has been shown that an earlier initiation of treatment usually provides a better therapeutic outcome [131]. Thus, it would be interesting if such a social platform could be effectively linked to new therapies.

## Figures and Tables

**Table 1:** mRNA-based immunotherapy in preclinical studies.

First Author	Year	Disease Type	Disease	Target	Method	Ref.
Magnani, CF	2023	Hematologic	AML	CD117	EP	[17]
Zhou, J-E	2022	Hematologic	ALL	CD3/CD19	LNP	[18]
Kavanagh, H	2021	Hematologic	ALL	CD19	Solupore non-viral delivery system	[19]
Parayath, NN	2020	Hematologic	ALL	CD19	PBAE polymer	[20]
Foster, JB	2019	Hematologic	ALL	CD19	EP	[21]
Köksal, H	2019	Hematologic	Lymphoma	CD37	EP	[22]
Panjwani, MK	2016	Hematologic	Lymphoma	Canine CD20z	EP	[23]
Almåsbak, H	2015	Hematologic	ALL	CD19	RV	[24]
Barrett, DM	2013	Hematologic	ALL	CD19	EP	[25]
Barrett, DM	2011	Hematologic	Leukemia	CD19	EP	[26]
Yang, Z	2023	Solid tumor	Hepatocellular carcinoma	CD24	LNP	[27]
Olivera, I	2023	Solid tumor	Melanoma	Gp75 (tyrosinase-related protein)	EP	[28]
Meister, H	2022	Solid tumor	Glioblastoma	NKG2D	EP	[29]
Subeh, ZYA	2022	Solid tumor	Melanoma	Fas	LNP	[30]
Lin, L	2021	Solid tumor	Myeloma	BCMA	EP	[31]
Kang M	2021	Solid tumor	Neuroblastoma	Mannose receptor	Polymer	[32]
Li, Z	2020	Solid tumor	Colorectal cancer	NKG2D	EP	[33]
Parayath, NN	2020	Solid tumor	Prostate tumor	ROR1	PBAE polymer	[20]
Parayath, NN	2020	Solid tumor	Hepatocellular carcinoma	HBcore18-27	PBAE polymer	[20]
Xiao, L	2019	Solid tumor	Colorectal cancer	NKG2D	EP	[32]
Hung, CF	2018	Solid tumor	Ovarian cancer	Mesothelin	EP	[34]
Ang, WX	2017	Solid tumor	Ovarian and colorectal cancer	Epithelial cell adhesion molecule	EP	[35]
Tchou, J	2017	Solid tumor	Breast and ovarian cancer	c-Met	EP	[36]
Caruso, HG	2016	Solid tumor	Glioblastoma	EGFR	EP	[37]
Krug, C	2015	Solid tumor	Melanoma	MCSP	EP	[38]
Schutsky, K	2015	Solid tumor	Ovarian cancer	Folate receptor alpha	EP	[39]
Singh, N	2014	Solid tumor	Neuroblastoma	Disialoganglioside GD2	EP	[40]
Lehner, M	2012	Solid tumor	Ewing’s sarcoma family of tumor	NKG2D	EP	[41]
Zhao, YB	2010	Solid tumor	Mesothelioma	Mesothelin	EP	[42]
Yoon, SH	2009	Solid tumor	Ovarian cancer	HER2	EP	[43]
Breda, L	2023	Congenital disorder	Sickle cell disease	CD117	LNP	[12]
Rurik, JG	2022	Congenital disorder	Cardiac fibrosis	CD5	LNP	[11]

EP, electroporation; PBAE, poly(β-amino ester).

**Table 2:** LNP-encapsulated base editors and prime editors in preclinical studies.

First Author	Year	Target Cells/Organ	Disease	Payloads *	Model	Editing Efficiency (%)	Ref.
Breda, L	2023	HSCs	Sickle cell disease	ABE8e	Mouse	>50	[12]
Palanki R	2023	Brain	Mucopolysaccharidosis type I	ABE7.10	Mouse	1.52–1.82	[69]
Musunuru K	2021	Liver	Hypercholesterolemia	ABE8.8	Monkey	64	[70]
Rothgangl T	2021	Liver	Hypercholesterolemia	ABEmax	Monkey	67	[71]
Rothgangl T	2021	Liver	Hypercholesterolemia	ABEmax	Mouse	34	[71]
Villiger L	2021	Hepatocyte	Phenylketonuria	CBE	Mouse	10.7	[72]

HSC, hematopoietic stem cells. * All include gRNA for payloads.

**Table 3:** U7 snRNA-mediated gene correction.

First Author	Year	Disease	Model	Vector	Mode of Action	Ref.
Almeida, CF	2023	Muscular dystrophy	In vitro	AAV8	Exon skipping	[91]
Wein, N	2022	DMD	Mouse	AAV9	Exon skipping	[92]
Monceau, A	2022	DMD	Mouse	AAV	Exon skipping	[93]
Wein, N	2021	DMD	Mouse	AAV9	Exon skipping	[88]
d’Arqom, A	2021	β-Thalassemia	Mouse	LV	Splicing correction	[94]
Aupy, P	2020	DMD	Mouse	AAV9	Exon skipping	[95]
Preedagasamzin, S	2018	β-Thalassemia	In vitro	LV	Splicing correction	[96]
Phanthong, P	2017	β-Thalassemia	In vitro	LV	Splicing correction	[97]
van der Wal, E	2017	Pompe	In vitro	LV	Exon inclusion	[90]
Odermatt, P	2016	SMA	Mouse	scAAV9	Exon inclusion	[98]
Nuzzo, F	2013	Factor V deficiency	In vitro	Plasmid/morpholino	Exon inclusion	[99]
Lorain, S	2012	Muscular dystrophy	Dog	AAV1	Exon skipping	[100]
Cecchini, S	2012	Muscular dystrophy	Dog	AAV6	Exon skipping	[101]
Goyenvalle, A	2011	DMD	In vitro; mouse	LV (In vitro); AAV (mouse)	Exon skipping	[102]
Voigt, T	2010	SMA	Mouse	LV	Exon inclusion	[89]
Geib, T	2009	SMA	In vitro	Adeno type 5	Exon inclusion	[103]
Goyenvalle, A	2009	DMD	In vitro	AAV	Exon skipping	[85]
Chaouch, S	2009	DMD	Mouse	LV	Exon skipping	[104]
Marquis, J	2009	β-Thalassemia	In vitro	LV	β-Thalassemia, splicing correction; SMN2, exon inclusion	[105]
Uchikawa, H	2007	PTCH, BRCA1, CYP11A	In vitro	Plasmid	PTCH, BRCA1; splicing correction; CYP11A; exon inclusion	[106]
Marquis, J	2007	SMA	In vitro	LV	Exon inclusion	[107]
Quenneville, SP	2007	DMD	Monkey	LV	Exon skipping	[108]
Denti, MA	2006	DMD	Mouse	AAV	Exon skipping	[109]
Madocsai, C	2005	SMA	In vitro	Plasmid	Exon inclusion	[110]
Goyenvalle, A	2004	DMD	Mouse	AAV	Exon skipping	[111]
Brun, C	2003	DMD	In vitro	Plasmid	Exon skipping	[112]
Vacek, MM	2003	β-Thalassemia	In vitro	LV	Splicing correction	[113]
Suter, D	1999	β-Thalassemia	In vitro	Plasmid	Splicing correction	[114]
Gorman, L	1998	β-Thalassemia	In vitro	Plasmid	Splicing correction	[115]

AAV, adeno-associated virus; DMD, Duchenne muscular dystrophy; LV, lentivirus; SMA, spinal muscular atrophy.

**Table 4:** ADAR-editable genes in preclinical studies.

First Author	Year	Gene	Model	Vector	Promoter	Ref.
Katrekar, D	2022	PCSK9	Mouse	AAV8	U6	[129]
Katrekar, D	2022	IDUA	Mouse	AAV8	U6	[129]
Yi, Z	2022	IDUA	Mouse	AAV8	Not described	[124]

ADAR, adenosine deaminase acting on RNA.
